# Quality of life in Montenegrin pupils with acne

**DOI:** 10.1371/journal.pone.0250155

**Published:** 2021-04-15

**Authors:** Milena Ražnatović Đurović, Milica Đurović, Janko Janković, Slavenka Janković

**Affiliations:** 1 Clinic of Dermatology and Venereology, Clinical Center of Montenegro, Podgorica, Montenegro; 2 Faculty of Medicine, University of Montenegro, Podgorica, Montenegro; 3 Institute of Social Medicine, Faculty of Medicine, University of Belgrade, Belgrade, Serbia; 4 Institute of Epidemiology, Faculty of Medicine, University of Belgrade, Belgrade, Serbia; Clinic for Infectious and tropical diseases, Clinical centre of Serbia, SERBIA

## Abstract

**Background:**

Acne is a common skin disease that can affect a person’s quality of life (QoL), self-esteem, and mood in an adverse manner. The aim of the current study was to assess QoL among Montenegrin pupils with acne.

**Methods:**

This cross-sectional survey was conducted over October and November 2020 in four randomly selected secondary schools in Podgorica, Montenegro. All 500 pupils were asked to fill in a short questionnaire which included questions on age, sex, presence of acne, and for those with acne their duration and location, visits to doctors, presence of any other coexisting skin disease, and family history of acne. Only pupils who self-reported acne were asked to complete the Children’s Dermatology Life Quality Index (CDLQI) and the Cardiff Acne Disability Index (CADI).

**Results:**

Self reported acne were presented in 49.8% (249/500) of all pupils. The mean CDLQI score of the total sample was 4.27 ± 5.13. Overall, the CDLQI domains that were most affected by acne were symptoms and feelings (mean score 1.49 ± 1.43), leisure (mean score 0.94 ± 1.72), and treatment (0.66 ± 0.79). The mean total CADI score was 3.53 ± 3.11 which was higher in girls (4.07 ± 3.11) than in boys (2.90 ± 3.00). There was good correlation between the two questionnaires (Rho = 0.76; P < 0.01). According to multiple linear regressions, higher overall CDLQI score was found in pupils with acne who reported other skin diseases, while girls, pupils who reported both acne on face and back, and who had any concomitant skin disease had higher CADI total score.

**Conclusions:**

Acne affects QoL of young adolescents in Montenegro with greater impact in girls. Our findings should point out the importance of timely diagnosis, treatment, and education of adolescents with acne.

## Introduction

Acne vulgaris, also known as acne, is one of the most common inflammatory dermatosis treated worldwide [1, 2]. The Global Burden of Disease study showed that acne was the 8th most prevalent disease globally in 2010 [3].

It is well known that acne is not just a trivial skin disease, but a psychosomatic disorder − a disease that involves both mind and body. Even mild to moderate disease can be associated with significant emotional and psychological issues such as depression and suicidal ideation often independent of the disease severity and greater than generally assumed [4].

Acne can affect people of all ages. Adolescents and young adults between ages 12 and 24 tend to be the most affected group [1]. About 90% of teenagers are affected by acne that usually occurs when adolescents are undergoing maximum physical and social transitions during a time of their low self-esteem and high concern about their physical appearance.

Numerous studies have confirmed that acne can negatively affect a person’s quality of life (QoL), self-esteem, and mood [5–12].

The present study aimed to assess health-related QoL among Montenegrin pupils with acne.

## Materials and methods

This cross-sectional survey was conducted in four randomly selected secondary schools in Podgorica, Montenegro. The sample size (500) was calculated using the assumption of 95% CI, and marginal error of 5%, enlarged with consideration on the loss of response. The head teacher of each school was approached and gave agreement for the study to be conducted during school-time. An explanatory letter describing the intended research was sent to the parents of pupils a week before the questionnaires were distributed to allow time for permission or refusal of their child’s participation in the study. Data were collected over October and November 2020. On the day of data collection a further explanation of the research was given to the pupils by medical doctor. Participation was voluntary and anonymous. All 500 pupils were asked to fill in a short questionnaire which included questions on age, sex, presence of acne, and for those with acne their duration and location, visits to doctors, presence of any other coexisting skin disease, and family history of acne. Only pupils who self-reported acne (n = 249) were asked to complete the Children’s Dermatology Life Quality Index (CDLQI) [13] and the Cardiff Acne Disability Index (CADI) [14].

The CDLQI is a skin disease specific 10-items questionnaire which covers all aspects of QoL: symptoms and feelings (two items), leisure time (three items), school or holidays (one item), personal relationships (two items), sleep (one item) and treatment (one item) in the previous week. The responses to questions are graded from 0 to 3, with a possible maximum score of 30 with higher scores representing more severely affected QoL [13]. The following severity bands for CDLQI scores were used in the interpretation of QoL: 0−1 no effect on QoL, 2−6 small effect, 7−12 moderate effect, 13−18 very large effect, 19−30 extremely large effect [15].

The CADI is a brief acne-specific questionnaire designed for measuring disability induced by acne. It consists of five questions relating to the previous months that cover feelings, symptoms, social life and perceived severity. Each question has four possible response categories (0–3) with a total maximum score of 15. The higher score means that more QoL is impaired [14].

Both questionnaires, the CDLQI and the CADI were linguistically validated and culturally adapted from English to Serbian language [16, 17].

The study was approved by the Ethics Committee of the Faculty of Medicine, University of Montenegro. The written informed consent was obtained from pupils’ parents.

### Statistical analysis

Categorical variables were expressed as counts and percentages while continuous variables were presented as mean ± standard deviation. The differences between variables were assessed by x^2^ or t-test. The Spearman Rho coefficient was used to examine the correlation between the CDLQI and the CADI questionnaires. Two multivariate logistic regression analyses were performed: the first one with the total CDLQI score as a dependent variable, and the second with the total CADI score as a dependent variable. Sex, disease duration, acne location, other skin disease, and family history of acne were independent variables in both analyses.

Statistical analysis was performed with the Statistical Package for the Social Sciences, SPSS version 20.0 (SPSS Inc., Chicago, IL, USA). A two-tailed probability value of 0.05 was considered significant.

## Results

Self reported acne were presented in 49.8% (249/500) of all pupils (115 boys and 134 girls). One hundred and ten boys and 116 girls reported acne alone. Nine percent (23/249) reported acne and coexisting skin disease, more girls (13.4%) than boys (4.3%). Face was the most dominant location (61.4%). Family history of acne was present in more than a half of all pupils (57.4%). Almost 80% of respondents with acne did not seek medical help (Table 1).

**Table 1:** Characteristics of study population.

Characteristic	Total sample (N = 249)	Boys (N = 115)	Girls (N = 134)	*P value*
Age, years (mean ± SD)	15.05 ± 0.51	15.16 ± 0.59	14.96 ± 0.40	0.002*
Disease duration				
Less than 1 year	91 (36.5%)	45 (39.1%)	46 (34.3%)	n.s.
More than 1 year	158 (63.5%)	70 (60.9%)	88 (65.7%)	
Acne location				
Only face	153 (61.4%)	72 (62.6%)	81 (60.4%)	n.s.
Only back	11 (4.4%)	3 (2.6%)	8 (6.0%)	
Face and back	85 (34.1%)	40 (34.8)	45 (33.6%)	
Other skin disease				
Yes	23 (9.2%)	5 (4.3%)	18 (13.4%)	0.028**
No	226 (90.8%)	110 (95.7%)	116 (86.6%)	
Family history of acne				
Yes	143 (57.4%)	66 (57.4%)	77 (57.5%)	n.s.
No	106 (42.6%)	49 (42.6%)	57 (42.5%)	
Visited doctor				
Yes	50 (20.1%)	18 (15.7%)	32 (23.9%)	n.s.
No	199 (79.9%)	97 (84.3%)	102 (76.1%)	

*t-test

**Chi-square test.

The overall CDLQI and CADI scores, and scores for CDLQI domains and CADI items, according to sex are presented in Table 2.

**Table 2:** Children’s Dermatology Life Quality Index (CDLQI) domains and Cardiff Acne Disability Index (CADI) according to sex (mean ± SD).

CDLQI	Total sample	Boys	Girls	P value*
Symptoms and feelings (items 1 and 2)	1.49 ± 1.43	1.41 ± 1.43	1.57 ± 1.43	n.s.
Personal relationships (items 3 and 8)	0.55 ± 1.07	0.51 ± 1.12	0.57 ± 1.04	n.s.
Leisure (items 4, 5 and 6)	0.94 ± 1.72	0.77 ± 1.56	1.09 ± 1.85	n.s.
School or holiday (item 7)	0.40 ± 0.73	0.39 ± 0.73	0.41 ± 0.73	n.s.
Sleep (item 9)	0.23 ± 0.60	0.22 ± 0.63	0.25 ± 0.57	n.s.
Treatment (item10)	0.66 ± 0.79	0.63 ± 0.78	0.69 ± 0.80	n.s.
Total score	4.27 ± 5.13	3.93 ± 5.24	4.57 ± 5.04	n.s.
CADI				
Feelings and symptoms (item 1)	0.78 ± 0.78	0.57 ± 0.73	0.96 ± 0.77	<0.0001
Personal relationships (item 2)	0.53 ± 0.78	0.43 ± 0.70	0.60 ± 0.83	n.s.
Avoidance of swimming (item 3)	0.31 ± 0.63	0.26 ± 0.62	0.36 ± 0.64	n.s.
Feelings about the appearance of skin (item 4)	0.94 ± 0.81	0.77 ± 0.82	1.10 ± 0.77	0.001
Perceived severity (item 5)	0.97 ± 0.75	0.87 ± 0.76	1.07 ± 0.73	0.045
Total score	3.53 ± 3.11	2.90 ± 3.00	4.07 ± 3.11	0.003

*t-test.

The mean CDLQI score of the total sample was 4.27 ± 5.13. Overall, the CDLQI domains that were most affected by acne were symptoms and feelings (mean score 1.49 ± 1.43), leisure (mean score 0.94 ± 1.72), and treatment (0.66 ± 0.79). There were no statistically significant differences between boys and girls neither in total CDLQI scores, nor in CDLQI domain scores (Table 2).

According to severity bands for CDLQI scores the effect of acne on QoL was small in most pupils (43.4%), moderate in 12.9, and large in 8.4%. More than one-third of pupils (35.3%) reported no effects on QoL.

The mean total CADI score was 3.53 ± 3.11 which was higher in girls (4.07 ± 3.11) than in boys (2.90 ± 3.00). There were also statistically significant differences between girls and boys in feelings and symptoms, feelings about the appearance of skin, and perceived severity with higher mean scores in girls (Table 2).

Sixty percent of pupils with acne in this study experienced symptoms and almost one-half (47%) was emotionally affected. Thirty eight percent of pupils were affected in their personal and social lives of whom 11% were moderately to severely affected. Sixty eight percent of all pupils with acne was concerned about the appearance of their skin of which 45% were occasionally concerned. However, 9 pupils (3.6%) felt very depressed and miserable. Even 24% of pupils avoided swimming because of their skin trouble. Eight percent of pupils felt their acne affected very much or quite a lot their school work and 13% of pupils had experienced bullying because of their acne. Acne-related sleep problems were reported by 16% of pupils and almost one-half of pupils (49%) reported problem because of the treatment for their acne. For more than one half of pupils (52.6%) their acne was a minor problem, while for 18.1% it was a major problem. However, a small but important minority (3%) were thought their acne was the worst it could possibly be.

Spearman’s correlation coefficients between the CDLQI domains and the CADI items ranged between 0.312 and 0.584. The highest correlations were seen between feelings about the appearance of skin and perceived severity (Rho = 0.722; P < 0.01), and symptoms and feelings and feelings about appearance of skin (Rho = 0.672; P < 0.01) for items of the CADI; and between symptoms and feelings and personal relationships (Rho = 0.553; P < 0.01) and leisure and treatment (Rho = 0.515; P < 0.01) for CDLQI domains (Table 3).

**Table 3:** Correlation matrix of Children’s Dermatology Life Quality Index (CDLQI) and Cardiff Acne Disability Index scores (CADI).

	CADI					CDLQI				
	CADI 1	CADI 2	CADI 3	CADI 4	CADI 5	SF	Leisure	S/H	PR	Sleep
CDLQI										
Treatment	0.515*	0.494*	0.341*	0.502*	0.548*	0.499*	0.515*	0.397*	0.512*	0.363*
Sleep	0.320*	0.312*	0.324*	0.330*	0.331*	0.413*	0.340*	0.342*	0.367*	
PR	0.463*	0.570*	0.518*	0.497*	0.466*	0.553*	0.466*	0.485*		
S/H	0.392*	0.348*	0.389*	0.406*	0.377*	0.486*	0.350*			
Leisure	0.478*	0.530*	0.423*	0.542*	0.505*	0.506*				
SF	0.496*	0.479*	0.451*	0.584*	0.552*					
CADI										
CADI 5	0.608*	0.530*	0.499*	0.722*						
CADI 4	0.672*	0.613*	0.528*							
CADI 3	0.431*	0.554*								
CADI 2	0.586*									

* P < 0.01 SF: Symptoms & feelings; S/H: School/holiday; PR: Personal relationships. CADI 1: Feelings and symptoms; CADI 2: Personal relationships; CADI 3: Avoidance of swimming; CADI 4: Feelings about the appearance of skin; CADI 5: Perceived severity.

There was a strong linear correlation between the mean scores of two questionnaires (Rho = 0.763; P < 0.01).

According to multiple linear regressions, higher overall CDLQI score was found in pupils with acne who reported other skin diseases (B = 4.792; CI = 2.601 − 6.983), while girls, pupils who reported both acne on face and back, and who had any concomitant skin disease (B = 2.374; CI = 1.052 − 3.642) had higher CADI total score (Table 4).

**Table 4:** Predictive value of characteristics of pupils with acne in relation to QoL measures.

Score	Characteristic	B	95% CI		*P*
CDLQI	Sex	0.199	-1.050	1.448	0.754
	Disease duration	-0.085	-1.440	1.270	0.902
	Acne location	0.678	-0.025	1.382	0.059
	Other skin disease	4.792	2.601	6.983	0.000
	Family history of acne	-0.410	-1.685	0.865	0.527
CADI	Sex	0.945	0.207	1.683	0.012
	Disease duration	0.304	-0.497	1.105	0.456
	Acne location	0.647	0.231	1.063	0.002
	Other skin disease	2.347	1.052	3.642	<0.0001
	Family history of acne	-0.047	-0.800	0.707	0.903

B: unstandardized regression coefficient; CDLQI: Children’s Dermatology Life Quality Index; CADI: Cardiff Acne Disability Index.

## Discussion

To our knowledge, similar research concerning impairment of health-related QoL in young adults with acne was not conducted in Montenegro. The prevalence of self-reported acne in the present study was about 50%, confirming previous findings that acne is common in adolescents. The frequency of acne in our study is similar to the findings in Serbian [18] and a Greek study [19], higher than in the Egyptian study [12], but significantly lower than in most other studies [5, 7–9, 12, 20, 21].

In this cross-sectional study, the QoL of Montenegrin pupils with acne was assessed using two instruments, the CDLQI, dermatology-specific, and CADI, acne-specific questionnaire. Although the overall mean scores for both the CDLQI (4.27; maximum score possible 30) and the CADI (3.53; maximum score possible 15) are pretty low, our study confirms that acne is associated with significant impairment in QoL. The reason for relatively low scores may be the predominance of clinically mild acne in the community setting.

Olsen et al. [22] in a meta-analysis related to the impact on the QoL of children’s skin conditions, reported that the overall estimated CDLQI score for acne (mean score 5.3) was in the sixth place after scabies (9.2), urticaria (7.1), atopic eczema (8.5), psoriasis (8.0), and vitiligo (6.5). The total CADI mean score (3.53) found in this study is in agreement with the results from previous studies conducted in Serbia [9, 23], somewhat higher than in the Scottish study [5], but significantly lower than in several other studies [12, 24, 25]. Sampogna et al. reported that the only skin diseases that had a greater psychosocial impact than acne were hyperhidrosis, hirsutism, ectoparasitic infections, and bullous diseases [26].

Although most pupils in this study were found to experience none or small and moderate effect of acne on QoL, important 8.4% of all pupils experienced large (6%) and extremely large effect (2.4%). The meta-analysis of studies where the distributions of CDLQI scores were provided found that 1–5% of children with acne experienced a very large impact on QoL [22].

In the present study, almost half (47%) of pupils were emotionally affected. The interaction of acne and psychosocial problems is complex and can cause negative emotional reactions, such as anxiety, helplessness and frustration, depression, and even suicidal ideation which can lead to impaired QoL and self-esteem [10, 27, 28]. Several previous studies also reported significantly increased psychological burden in acne patients [29–33]. Pawin et al. [6] reported that 58.2% of patients with acne felt lonely, and 56.5% felt anxious. The data from national survey of youth health conducted in New Zealand (Youth 2000), pointed out that acne was associated with an increased probability of depressive symptoms, anxiety, and suicide attempts [34]. Our study suggests that young people presenting with acne are vulnerable to psychological problems. Therefore, it is important to detect such problems early and offer effective therapy to prevent their progression.

Adolescence is an important period, both for identity and social development. In this study, 38% of pupils were affected in their personal and social lives of whom 11% were moderately to severely affected. In the French study, nearly half of acne patients (48%) reported that their daily lives were affected [6]. It is important to identify and treat teenagers with acne early to reduce the future socio-economic burden of their acne. Pupils need to be informed that acne can be treated.

In our study girls were reported to experience a greater impact on QoL than boys although statistically significant difference was seen only for the CADI total score and for the CADI items feelings and symptoms, and feelings about the appearance of skin. This indicates that the psychological impact of acne may be greater for females than it is for males. Similarly, several studies found that girls are more susceptible than boys to the negative psychological effects of acne [9, 35–40]. However, Greek and Egyptian authors did not find any difference in QoL between male and female adolescents affected with acne [10, 12].

Our finding that pupils with coexisting skin diseases had more impaired QoL is in line with the finding from a Serbian study of 478 pupils of the secondary railway-technical school [9].

Only a few studies analyzed the association between family history and QoL in patients with acne. Guo et al. [41] reported that patients with skin diseases (including acne) with a family history, were more likely to affect the QoL for 2.221 (95% CI 1.333–3.703) times than those without a family history. The possibility of acne affecting QoL in the same study was 1.219 (95% CI 0.589–2.520) times that of patients with psoriasis. Indian authors found a statistically significant correlation between family history and DLQI/CADI scores [42] and concluded that family history played a role in affecting the QoL. In the present study, we did not find any statistically significant association between family history of acne and CDLQI/CADI scores.

The impact of duration of acne on QoL was analyzed in several studies. In a Brazilian study subjects with shorter acne duration presented significantly higher scores of the Acne-Specific Quality of Life Questionnaire (Acne-QoL), i.e. better QoL [43]. In contrast, Tan et al. [40] reported that a greater impact on QoL, evaluated with the same questionnaire (Acne-QoL), was associated with longer acne duration. We did not find any significant correlation between acne duration and QoL.

Our study confirmed a strong correlation between the CDLQI and CADI questionnaires. Pupils who scored highly on the CDLQI also tended to score highly on the CADI which is in agreement with previous studies [5, 9].

The strength of the present study was a representative sample of young adolescents surveyed from the general population. However, cross sectional design of study may introduce biases associated with self-reporting such as misclassification bias and under- or over-reporting of information. In addition, QoL instruments used in this study are not sufficient to diagnose psychological disorders without clinical assessment through interview of the affected pupils.

Nevertheless, despite these limitations, the current study confirms the negative impact of acne on the QoL of Montenegrin teenagers, and the high psychosocial burden in some of them. Therefore, we recommend that more attention should be paid to the psychological problems of the pupils with acne, to detect them early and offer effective therapy.

This study also has shown a strong correlation between the CDLQI and CADI. In addition, both QoL instruments were easily understood and quickly completed by pupils with acne.

## Supporting information

S1 File Dataset. (XLS)
